# Randomised trial on the economic impact of proficiency‐based progression vs conventional robotic surgical training

**DOI:** 10.1111/bju.70130

**Published:** 2026-01-02

**Authors:** Stefano Puliatti, Natali Rodriguez Peñaranda, Marco Amato, Ruben De Groote, Rui Farinha, Brendan Bunting, Ben van Cleynenbreugel, Alexandre Mottrie, Anthony G. Gallagher

**Affiliations:** ^1^ University of Modena and Reggio Emilia Modena Italy; ^2^ Department of Urology Azienda Ospedaliero‐Universitaria di Modena Modena Italy; ^3^ Department of Urology Onze‐Lieve‐Vrouw Hospital Aalst Belgium; ^4^ Orsi Academy Melle Belgium; ^5^ Department of Development and Regeneration KU Leuven Leuven Belgium; ^6^ Centro Hospitalar Universitário de Lisboa Central EPE Lisbon Portugal; ^7^ School of Psychology Ulster University Coleraine UK; ^8^ School of Medicine, Faculty of Life and Health Sciences Ulster University Londonderry UK

**Keywords:** cost effectiveness, proficiency‐based progression, Randomised trial, robotic surgery, surgical training

## Abstract

**Objective:**

To evaluate the cost‐effectiveness of proficiency‐based progression (PBP) training compared to conventional surgical training approaches, and to determine whether PBP training implementation is economically justified when scaled to large numbers of trainees.

**Methods:**

Economic analysis was performed using data from the prospective, randomised, and blinded Orsi Surgical Skills E‐learning Trial (OSSET; ClinicalTrials.gov identifier: NCT04541615) at ORSI Academy (Belgium), where 47 medical trainees without prior robotic surgery experience were randomised into four groups, each with progressively reduced adherence to the PBP methodology. All trainees completed simulation‐based training on a validated bladder‐urethra anastomosis model, ranging from full PBP training with metric‐based assessment and proficiency benchmarks (Group 1) to a traditional apprenticeship model (Group 4). The primary outcome was training cost, evaluated per trainee and based on programme scalability (12–500 trainees), including expenses for accommodation, laboratory time, and metric development. Cost equivalence points and scalability thresholds were identified to compare the financial impact of the four training strategies.

**Results:**

The PBP training was more expensive than conventional methods for small cohorts (e.g. €14 139 vs €7067 per trainee for 12 trainees), but became significantly more cost‐effective beyond 25 trainees (equivalence point). At 500 trainees, total PBP training cost was €1.69 million compared to €3.53 million for conventional training, a 110% cost advantage. All differences were statistically significant (*P* < 0.001).

**Conclusions:**

We conclude that PBP training is significantly more effective and becomes increasingly cost‐efficient as the number of trainees increases. These findings support its integration into high‐volume national training programmes, offering a scalable and economically sustainable alternative to apprenticeship‐based surgical education.

**Abbreviations**
ASA: American Surgical Association
ERUS: European Association of Urology Robotic Urology Section
IRR: inter‐rater reliability
OSSET: Orsi Surgical Skills E‐learning Trial
PBP: proficiency‐based progression

## Introduction

Proficiency‐based progression (PBP) is an innovative surgical training paradigm that uses objective metrics to ensure trainees reach a predefined proficiency benchmark based on expert performance [1]. Studies have shown PBP to be more effective than traditional methods [2], with a systematic review by Mazzone et al. [3] finding that PBP training led to a 60% improvement in performance compared to conventional approaches. The European Association of Urology Robotic Urology Section (ERUS) was the first professional society to adopt a PBP‐based training curriculum for robotic procedures, including the ERUS robot‐assisted radical prostatectomy programme, which integrates theoretical education, preclinical and clinical training, and final evaluation [4]. One of its key components is urethro‐vesical anastomosis training, for which the chicken model is a widely used and cost‐effective simulation tool [4]. A previous prospective randomised trial by Puliatti et al. [5] further demonstrated the efficiency and effectiveness of PBP training, showing that 100% of PBP trainees achieved proficiency, compared to 58% in the conventionally trained group. As adherence to PBP methods decreased, the efficiency of reaching proficiency significantly declined [5].

Despite its proven benefits, there are concerns in the surgical community about the cost of metric development and validation that underpin the PBP training methodology. Furthermore, the economic impact of PBP training remains unexplored, with no cost analysis conducted in any surgical field where PBP training methods have been applied. This is an important aspect, given the rapidly increasing number of surgical residents needing general training, and, even more so, robotic surgical training. The number of residents requiring general surgical training has been estimated at approximately 2000 annually in the United States for general surgery alone [6], while major European robotic training centres, such as the ORSI Academy in Belgium, report training approximately 2000 trainees per year [7]. Reproducible, efficient and quality‐assured educational models are increasingly necessary to meet the expanding demand for high‐quality robotic surgical education. Despite its higher upfront investment (i.e. in metric development and validation), PBP training may offer a significant return on investment, particularly when applied to larger cohorts. The current study evaluates the economic implications of PBP‐based curricula compared to other training methodologies. The aim was to determine whether the benefits of such curricula justify their broader implementation in surgical education systems. Specifically, using the results from Puliatti et al. [5], we compared the costs of four training methods, each representing a progressively reduced adherence to the PBP model. We hypothesised that, despite higher initial costs, a complete PBP training programme could lead to significant cost savings over the long term and when implemented on a large scale, compared to other training methodologies.

## Methods

### Study Design and Participants

**Fig. 1:**
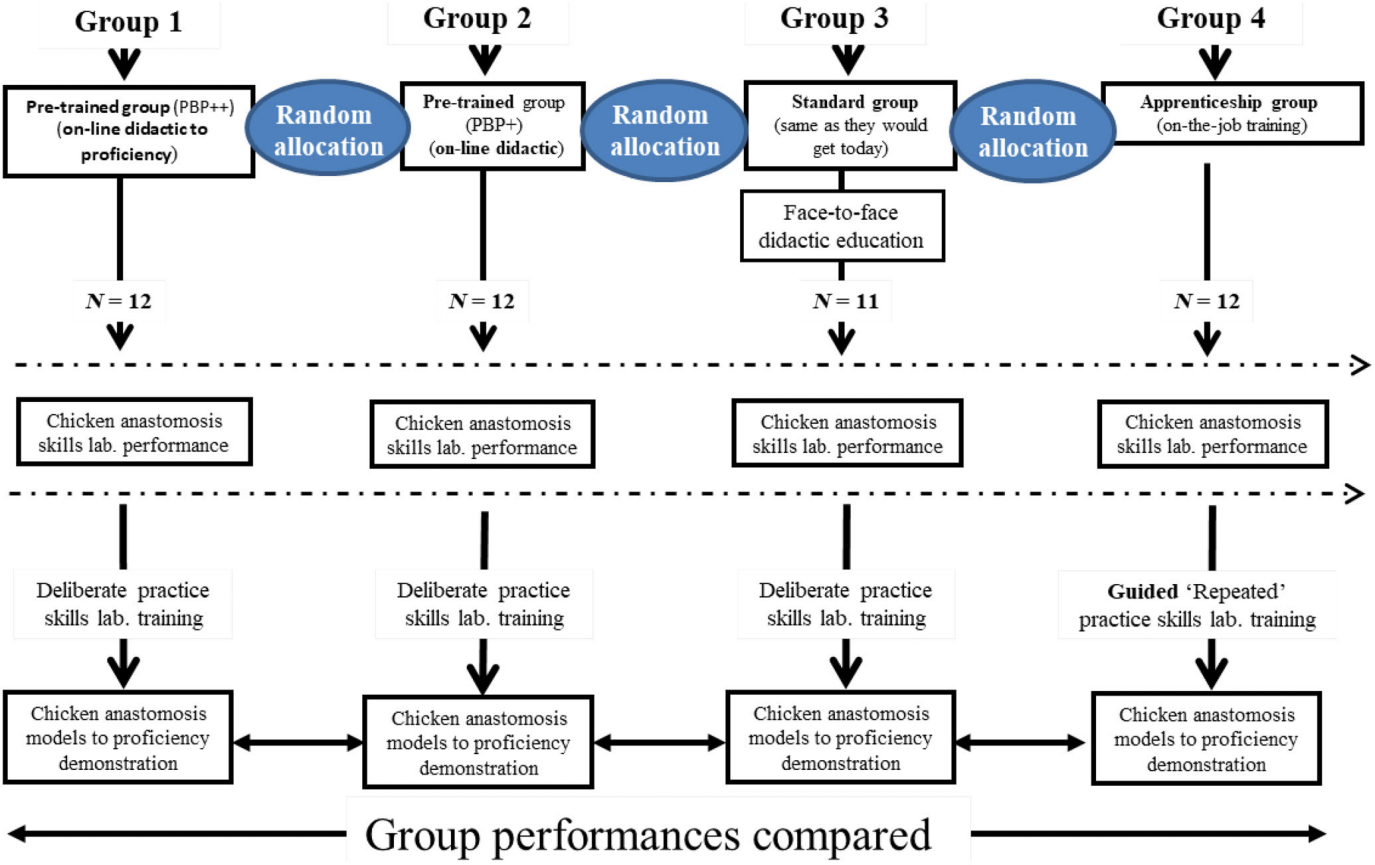
The study design for the prospective, randomised, training and objective assessment for between‐group performance of chicken anastomosis suturing and knot‐tying performance. lab., laboratory; PBP, proficiency‐based progression.

The current study is based on a previously published prospective study conducted at the ORSI Academy (Belgium), with expedited institutional review board approval from OLV Hospital, Aalst, Belgium (approval 22 July 2020), and registration on ClinicalTrials.gov (NCT04541615; Fig. S1 shows the CONSORT flow diagram) [5]. The study included 48 Belgian medical students with an interest in robotic surgery (Table 1). As shown in Fig. 1, participants were randomly assigned using RANDOM.ORG (1:1:1:1 ratio). Each group (12 participants each) received different didactic preparation before practical training:

- Group 1 (Full PBP): online training with mandatory proficiency benchmark before practice;
- Group 2 (eLearning): same online content, but no benchmark required;
- Group 3 (Traditional): in‐person lectures with free access to practice;
- Group 4 (Apprenticeship): self‐study using peer‐reviewed papers, without structured instruction or benchmarks.

**Table 1:** Demographic data, personal characteristics and data on the surgical experience of the participants.

	Group 1	Group 2	Group 3	Group 4	Total
Number of participants	12	12	11	12	47
**Gender, *n* (%)**					*N* (%)
Male	6 (13)	5 (11)	4 (8)	6 (13)	21 (45)
Female	6 (13)	7 (15)	7 (15)	6 (13)	26 (55)
**Sight correction, *n* (%)**					
Yes	5 (11)	6 (13)	7 (15)	4 (8)	22 (47)
No	7 (15)	6 (13)	4 (8)	8 (17)	25 (53)
**Surgical robotic courses attended, *n* (%)**					
Yes	0 (0)	1 (2)	0 (0)	1 (2)	2 (4)
No	12 (26)	11 (23)	11 (23)	11 (23)	45 (96)

All groups underwent the same assessment at the end of the theoretical module, but only Group 1 had to meet a benchmark before proceeding to practical training.

### Practical Robotic Training

The study evaluated robotic suturing and knot‐tying performance through a validated and metricised bladder‐urethra anastomosis on a chicken simulation model [8]. Three trainees were assigned per surgical robotic system to train on the suturing and knot‐tying task. Each trainee trained for a maximum of 30 min to perform the task, during which their performance was scored in real time by a trainer. The trainer, trained in scoring the metrics, provided coaching and constructive formative feedback. If a trainee failed to meet the proficiency benchmark within the allotted time (30 min), the session ended to allow the next trainee to perform the task. As a result, it took 90 min for all three trainees to complete one training trial. Prior to the training session on the Da Vinci Si/X/Xi system, all participants completed robotic simulation on the da Vinci® Skills Simulator (DVSS). Performances were recorded and evaluated by blinded expert raters. Training continued until benchmarks were met. Groups 1–3 received performance metrics, while Group 4 was guided by experienced robotic surgeons (>10 years’ experience). Training was limited to 3.5 days per group.

Study‐specific details have previously been reported by our group in a study on the effectiveness of PBP training [5]. Trainees were batched in groups of 12 trainees per group. The PBP trainers did not know the group allocation. Trainers knew that they would be delivering the same PBP training to all trainees. The only difference for the trainees was how they were prepared BEFORE they went into the skills laboratory. The trainers for the conventional training group did know that they would be delivering conventional training. We also explained to them that we were comparing conventional training from surgeons who were reputed to be good surgeons and good trainers to ‘other’ approaches to training. Furthermore, the conventional group trainers were all experienced and practising surgeons. All of the PBP trainers were much more junior. The PBP trainers had all completed a Urology Fellowship programme at ORSI Academy.

### Assessments

In the PBP methodology all of the raters are educated to understand the metrics and then trained to score the same video with the metrics until they demonstrate an inter‐rater reliability (IRR) of >0.8. Then, pairs of raters, individually, score different videos. Score patterns are then compared for each pair immediately after video scores were completed. Differences in score allocation are scrutinised and discussed whilst viewing the video and reasons for different score patterns elucidated. This process continues until the pair of raters demonstrate an IRR >0.8, consistently (i.e. more than two times consecutively). The vast majority of raters take only two to three videos to achieve an IRR >0.8 consistently. Only at this point are scorers permitted to assess the study videos.

### Cost Analysis

The cost calculations in the current study were derived from data extrapolated from the Orsi Surgical Skills E‐learning Trial (OSSET) [5]. For this analysis, specific cost components were assigned to various aspects of the training programme:

- Hotel accommodation: standardised at €200 per night per trainee, included for all groups.
- Laboratory costs: €2450 per person per day (only full or half days were considered). Applied across all groups.
- Additional costs for Groups 1–3: these groups incurred extra costs related to metric development, validation, and eLearning materials, totalling €132 377, which was evenly distributed among trainees, for example:
  ○ 12 trainees: €11 031 per trainee;
  ○ 50 trainees: €2648 per trainee;
  ○ 100 trainees: €1324 per trainee;
  ○ 200 trainees: €662 per trainee;
  ○ 500 trainees: €265 per trainee.
- Metrics cost allocation: exclusive to Groups 1–3 and not applied to Group 4.
- Group 4 costs: included only hotel accommodation and laboratory time.

Costs were calculated for each individual trainee based on the number of trials required to reach the proficiency benchmark, the number of full or half days spent in the robotic surgery skills laboratory, and the number of nights stayed in the hotel.

### Statistical Analysis

Differences in training performance (e.g. time to proficiency) were assessed using SPSS v29, (Chicago, IL, USA). Cost comparisons were based on modelled scenarios using extrapolated data from the OSSET study to assess whether the higher initial investment associated with PBP translates into long‐term savings compared to traditional methods [5]. We used the principles of Markov models to predict costs of training larger numbers of trainees with information we have gathered from the OSSET study. A Markov model is a statistical model used to predict the behaviour of a system over time, based on a set of discrete states and the probabilities of transitioning between them. The core principle, known as the Markov property, is that the future state depends only on the current state. We therefore had reliable information on: (1) the number of training trials it took trainees to reach proficiency with the different training methodologies; (2) the number of training days in the skills laboratory; (3) the number of nights in a hotel; (4) the cost of lecture room hire; and (5) the costs of metric development and validation. Costs from Factors 1–4 were determined by the duration of training. The costs of the metrics (Factor 5) were a fixed sum (i.e. €132 377). However, when these fixed costs were distributed across an increasing number of trainees, the per‐trainee cost decreased substantially  (e.g. €11 031 per trainee for 12 trainees versus €265 per trainee for 500 trainees). We used the knowns for Factors 1–4, which were known and depended on time to proficiency demonstration, as well at the cost of the metrics, which depended on the number of surgeons being trained. This information was then used to predict the cost per trainee and total cost for training 12, 50, 100, 200, 300, 400 and 500 trainees – in cohorts of 12 trainees. It was further assumed that trainees would be trained under the same conditions as described in the OSSET study.

## Results

### Participant Characteristics

A total of 48 Belgian medical students with an interest in robotic surgery were enrolled and randomised equally into four groups (12 trainees). One withdrawal in Group 3 resulted in a final cohort of 47 participants (Table 1). The median age was 22 years. Overall, 21 participants (45%) were male and 26 (55%) female. Nearly half of the cohort (47%) required sight correction. Only two participants (4%) had previously attended a surgical robotic course, while the vast majority (96%) had no prior robotic training experience. There were no statistically significant differences between groups for these demographic and experiential variables.

### Learning Performance and Efficiency across the Four Studied Groups

**Fig. 2:**
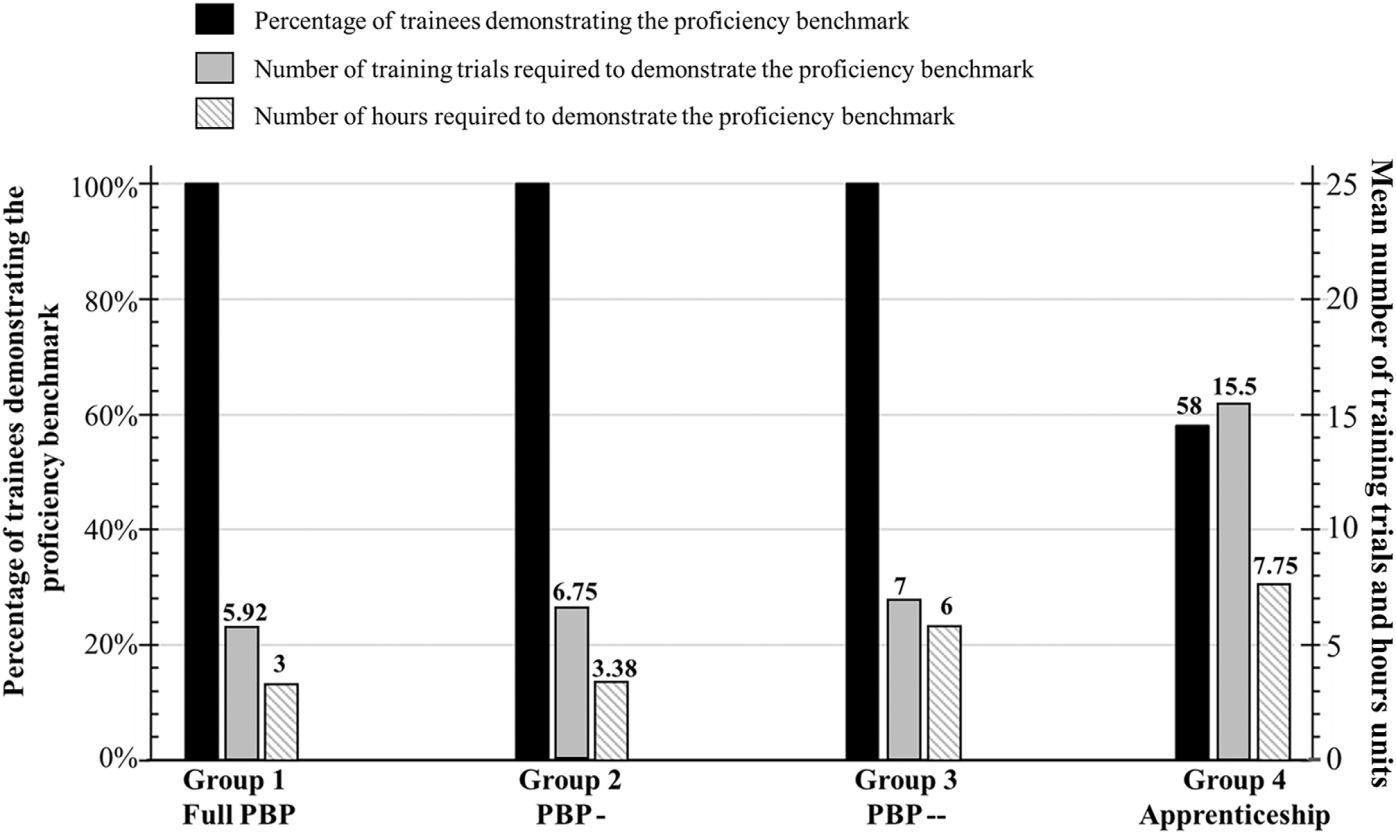
A summary of the performance outcomes for Groups 1–4 on the chicken anastomosis suturing and knot‐tying performance for percentage of trainees who demonstrated the proficiency benchmark, the mean number of training trials to reach proficiency and the mean robotic surgery hands‐on training time. PBP, proficiency‐based progression.

All groups improved after training, but the degree of improvement varied. The more the training deviated from full PBP methodology, the more errors and attempts were needed to reach the proficiency benchmark. As shown in Fig. 2, Groups 1–3 (PBP‐based) achieved 100% proficiency, while only 58% of Group 4 (Apprenticeship) reached the benchmark. Group 1 (Full PBP) required the fewest attempts (mean 5.92 training trials), compared to Group 4, who required a mean of 15 training trials. Training time followed the same pattern:

- Group 1 needed a mean of 2.96 h to reach the proficiency benchmark, which translated into a median of 1 full day in the skills laboratory (mean 1.17 days).
- Group 2 required a mean of 3.38 h (+14%) to reach the benchmark, which translated into a median of 1.25 full days in the skills laboratory (mean 1.29 days)
- Group 3 required a mean of 6.00 h (+103%) to reach the benchmark, which translated into a median of 1.5 full days in the skills laboratory (mean 1.5 days)
- Group 4 required a mean of 7.75 h (+162%) to reach the benchmark, which translated into a median of 2.5 full days in the skills laboratory (mean 2.67 days)

### Cost of PBP Training Compared to Conventional Training: Per Trainee

**Fig. 3:**
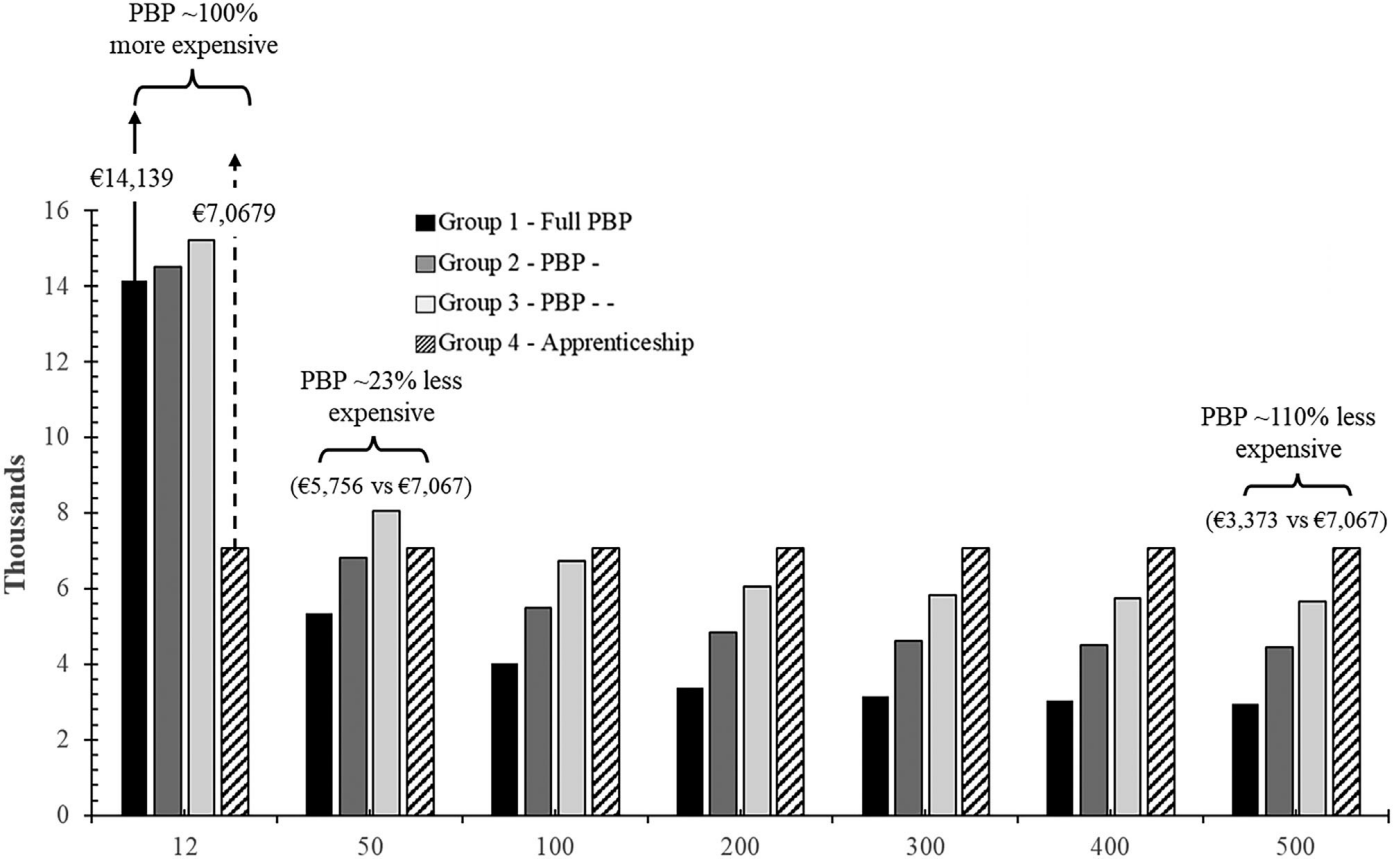
Cost of proficiency‐based progression (PBP) training compared to conventional training – per trainee, for 12, 50, 100 and 500 trainees.

Figure 3 shows how the cost per trainee varied depending on both the training model and the number of participants. For small cohorts of 12 trainees, Group 1 training was substantially more expensive (€14 139 per trainee [95% CI 13 708–14 571]), nearly 100% more than Group 4 (€7067 [95% CI 6380–7754]). As cohort size increased, however, the cost per trainee in Group 1 decreased: €5756 (95% CI 5325–6188) per trainee for 50 trainees (~23% less than Group 4, which was €7067 [95% CI 6380–7754]). The average cost per trainee in Group 1 continued to decrease as the number of trainees increased. The average training cost for Group 1 for 500 trainees was €3373 (95% CI 2942–3805) in comparison to €7067 (95% CI 6380–7754) for Group 4. This meant that, when a full PBP approach (Group 1) was used for training 500 trainees, it was ~110% less expensive than the cost for the conventional apprenticeship (Group 4) approach to training.

### Total Cost of PBP Training Compared to Conventional Training

**Fig. 4:**
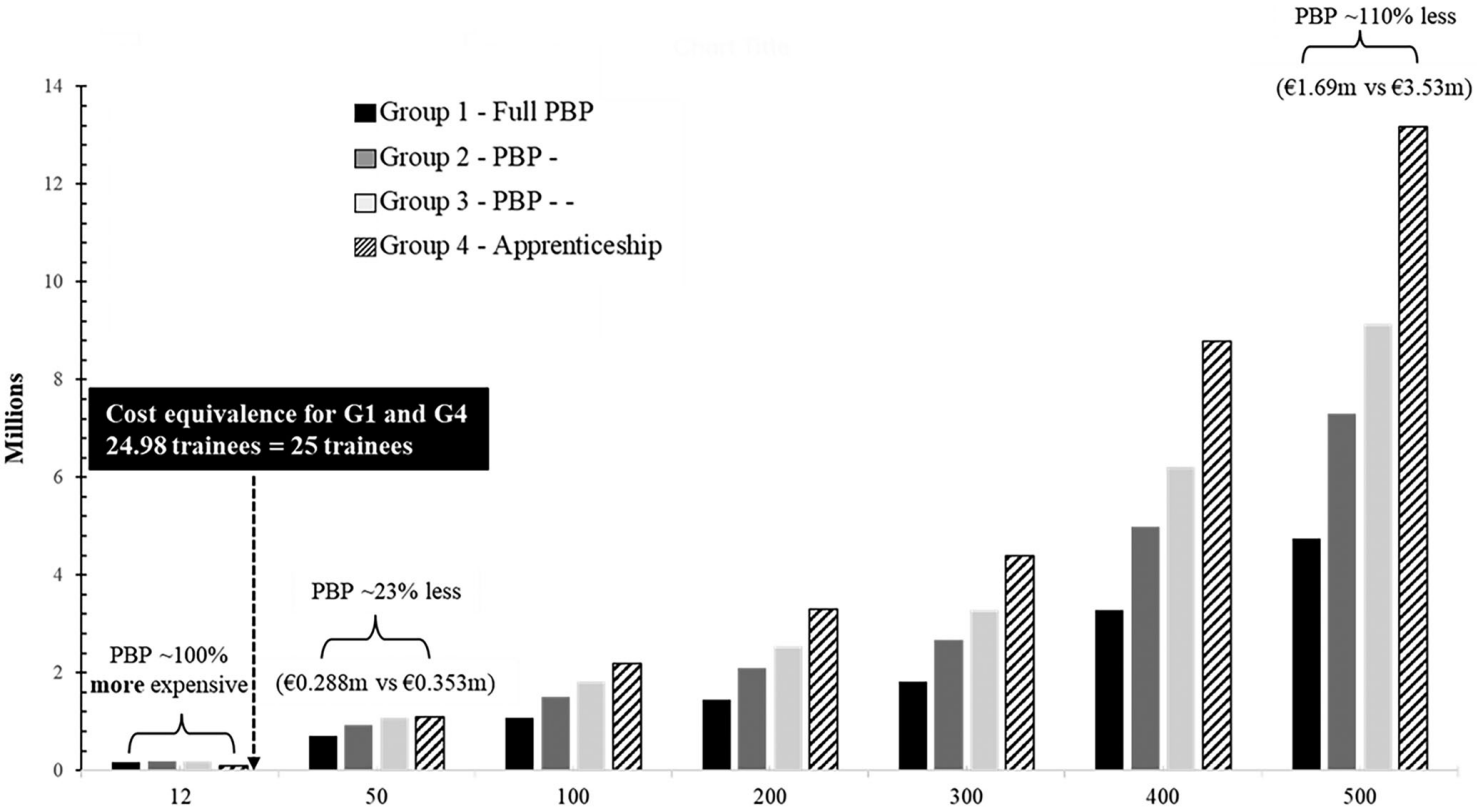
Total cost of proficiency‐based progression (PBP) training compared to conventional training, for 12, 50, 100 and 500 trainees.

Figure 4 shows how the total training cost varied depending on both the training model and the number of participants. For small cohorts of 12 trainees, Groups 1–3 had the highest total costs at €169 668 for Group 1, followed by Group 2 at €174 144, and Group 3 at €182 604. Group 4, at €84 804, was the least expensive for 12 trainees. For the 12 trainees scenario, Group 1 training was approximately 100% more expensive than Group 4. As the number of trainees increased, however, total training costs in Groups 1–3 grew at a slower rate. Specifically, for 50 trainees, total costs were €287 817 (95% CI 266 245–309 388) for Group 1, €306 462 (95% CI 277 849–335 076) for Group 2, €341 718 (95% CI 308 045–375 391) for Group 3, and €353 333 (95% CI 318 977–387 690) for Group 4. For 100 trainees, costs were €443 233 (95% CI 400 091–486 376) for Group 1, €480 525 (95% CI 423 297–537 752) for Group 2, and €551 036 (95% CI 423 297–537 752) for Group 3. The cost for 100 trainees in Group 4 was the highest, at €545 245 (95% CI 637 953–775 380). The cost equivalence point between full PBP (Group 1) and apprenticeship training (Group 4) was reached at 24.98 trainees. Beyond this threshold, Group 1 became increasingly cost‐effective. For training 500 surgeons, Group 1 training totalled €1 686 666 million (95% CI 1 470 954–1 902 379), while costs for Group 4 rose to €3 533 333 million (95% CI 3 189 766–3 876 901), a 110% cost saving in favour of PBP. Similar trends were observed for Groups 2 and 3 but differences were not as large as the comparison with Group 1.

## Discussion

The results from this study demonstrate that PBP training methods are a more cost‐effective approach to training >25 trainees in robotic surgical skills. We showed that the PBP approach is more costly with small numbers of trainees, namely, 12 trainees, in comparison to a conventional apprenticeship approach to training. As the number of trainees increase, the costs associated with a PBP approach reduce dramatically and very quickly the conventional apprenticeship approach to training becomes significantly more expensive. Furthermore, the calculations in this study are based on the assumption that 100% of the trainees receiving the conventional apprenticeship approach to training reached the proficiency benchmark. The reality is that, in the OSSET study [5], only 58% of participants reached the proficiency benchmark. Had this been factored into our calculations, the difference would have been even larger.

The first study to report the effectiveness of a PBP approach to training in surgical skills was reported in 2002 at the American Surgical Association (ASA) and has subsequently become a citation classic in the domain of surgical training using simulation methodology [9]. However, since the first reports of a PBP approach to training, some concerns have been raised by surgical leadership about its use in surgical education. One of the first major concerns was that the proficiency benchmark was too high for trainees to reach. Studies using a PBP methodology have consistently demonstrated that the vast majority of trainees do in fact reach the proficiency benchmark during the normal duration of a training course [1, 10, 11, 12]. Another concern, was whether all of the trainees could reach the proficiency benchmark. The OSSET [5] study by Puliatti et al. was the first prospective, randomised and blinded study that set out to establish whether all trainees could be trained to the proficiency benchmark. In that study, the authors very clearly demonstrated that all participants trained using variants of the PBP methodology could reach the proficiency benchmark. One of the surprising findings of the OSSET study was that only 58% of the trainees in the apprenticeship model achieved the proficiency benchmark, despite receiving nearly 3 full days of skills laboratory training with expert robotic surgeons known for their teaching excellence.

In their systematic review and meta‐analysis, Mazzone et al. included all prospective, randomised studies comparing performance outcomes between PBP and quality‐assured conventional training methodologies. Their analysis demonstrated that PBP trainees performed significantly better (i.e., ~60%) than those who received conventional training [3]. Furthermore, additional studies showed that a PBP approach to training is both more efficient and more effective than conventional methods [5, 11]. Despite this growing body of evidence supporting the superior efficiency and effectiveness of a PBP training method, concerns have been raised regarding the potential costs associated with implementing a PBP approach to surgical training. Specifically, critics have pointed to the potentially higher costs associated with the development and validation of objective performance metrics, as well as the longer training time required to ensure that all trainees reach a predefined proficiency benchmark. The present study represents the first systematic assessment of the actual costs of a PBP training model. Both the performance metrics and the PBP training approach were previously developed, implemented, and pubblished by the same research group, whereas the present work adds the final component by systematically evaluating the associated costs of both aspects.

The current results regarding the cost of PBP training per trainee show that the cost of training using a PBP methodology is only more expensive than conventional training if small numbers of robotic surgeons are to be trained. The cost equivalence point is reached at ~25 trainees, where the cost of PBP matches that of a conventional training approach. Beyond this threshold, PBP becomes the less expensive option. Therefore, the greater the number of trainees undergoing PBP training, the more cost‐effective the programme becomes. This is primarily driven by the superior efficiency and effectiveness of the PBP approach. All trainees in the PBP groups reached the proficiency benchmark within a single day of training, whereas only 58% of those in the conventional training group achieved the same benchmark after 3 full days in the skills laboratory.

It might be argued that the task used in this study was relatively simple and straightforward and that the results could have been different had a more complex procedure been employed. At odds with this assumption are findings from other PBP‐based studies. Specifically, in a cardiac resynchronisation therapy study involving a 2‐day, 3‐lead protocol, 93% of cardiology trainees in the PBP group reached the proficiency benchmark, compared to 0% in the control group [12]. Similarly, in a 2‐day course on arthroscopic procedures, 89% of trainees reached the benchmark for a Bankart repair, and 83% for a rotator cuff repair [11].

Our results regarding the total cost of the PBP training show that, while PBP training is initially more expensive for small cohorts (nearly 100% more costly than conventional training for 12 trainees), the cost per trainee decreases significantly as the number of participants increases. Specifically, PBP training becomes ~23% cheaper at 50 trainees, ~59% cheaper at 100 trainees, and up to ~110% cheaper at 500 trainees, compared to the apprenticeship model (Group 4). These findings indicate that a PBP approach becomes increasingly cost‐effective when applied at scale, making it particularly well suited for national training programmes involving large numbers of surgical trainees, such as the estimated 2000 new trainees per year in the United States and the European Union [6, 7]. In such programmes, the use of shared metrics, standardised proficiency benchmarks and metric‐based deliberate practice would allow for consistent implementation across multiple centres [2]. A PBP approach would thus underpin a truly standardised approach to simulation‐based training. This scale of training would underpin a very cost‐effective approach to the development and use of metrics. Furthermore, a more standardised, evidence‐based and validated approach to skills training may help to address the discontinuity crisis in medical education and training [13].

Lastly, it is now well established that intra‐operative surgeon performance has a significant impact on patient outcomes [14, 15]. This imperative will not be addressed by offering ‘more training’, but rather through better, evidence‐based training approaches that have been demonstrated to be effective [13]. A PBP approach to training has been demonstrated to significantly improve intra‐operative performance on real patients [9, 16, 17, 18], and importantly, to have a measurable impact on patient outcomes as well [19].

Despite its novelty, the current study is not without limitations. The data presented are based on a study with a relatively small number of participants. However, science relies on the clear demonstration of cause‐and‐effect relationships, and in this case, the observed effects (both in this study and in prior PBP vs conventional training comparisons) are large and supported by Level 1a evidence from a systematic review and meta‐analysis. Some debate may arise regarding the costs related to the skills laboratory or hotel accommodation. However, these were the actual costs at the time the study was completed and were not estimates. One cost not included in our analysis was the additional personal expenses incurred by trainees during their stay. This omission likely results in a conservative estimate, particularly for the apprenticeship training group, whose participants stayed for 3 nights, compared to only 1 night for Group 1. Moreover, the study was conducted in a single high‐volume training centre (ORSI Academy), which may limit the generalisability of the results to other training environments with different resources, faculty expertise, or infrastructure. However, the use of validated performance metrics, standardised tasks, and objective proficiency benchmarks helps ensure that the findings are transferable and reproducible across other centres adopting a similar proficiency‐based approach.

In conclusion, this study is the first to systematically assess the costs of PBP surgical training. While initially more expensive for small cohorts, PBP becomes more cost‐effective beyond 25 trainees, while also delivering superior training outcomes. All PBP trainees reached proficiency within 1 day, compared to 58% in the conventional group after 3 days. These findings support PBP as an efficient, scalable, and economically viable model for high‐volume surgical training programmes.

## Disclosure of Interests

Alexandre Mottrie is the CEO and a shareholder of ORSI Academy. Anthony G. Gallagher (AGG) was Director of Research and Education at ORSI at the time of the study but, other than supervising the scientific aspects, had no involvement in the training. AGG originally developed the PBP methodology and first reported it in 2002 at the ASA meeting, where it was presented as the ASA Educational Paper. The remaining authors have no conflicts of interest to declare.

## Supporting information


**Fig. S1.** CONSORT 2025 Flow Diagram. Flow diagram of the progress through the phases of a randomized trial of two groups (that is, enrolment, intervention allocation, follow‐up, and data analysis).
