# Supplementary material for: Randomised trial on the economic impact of proficiency‐based progression vs conventional robotic surgical training
Source: BJU Int. 2026 Jan 2;137(3):493–501. doi: 10.1111/bju.70130 (PMC12907777; doi:10.1111/bju.70130)
Supplement: Supplementary file 1 — Fig. S1. CONSORT 2025 Flow Diagram. Flow diagram of the progress through the phases of a randomized trial of two groups (that is, enrolment, intervention allocation, follow‐up, and data analysis). [file BJU-137-493-s001.docx]

**Supplementary Figure 1: CONSORT 2025 Flow Diagram**

Flow diagram of the progress through the phases of a randomised trial of two groups (that is, enrolment, intervention allocation, follow-up, and data analysis)

Blinded Assessment by 5-pairs of surgeon raters blinded to trainee and training group

Analysis

Traditional training to proficiency

PBP training to proficiency

GROUP 4: Traditional training + Lecture (n= 12)

GROUP 3: PBP training + Lecture (n= 11)

GROUP 2: PBP training + eLearning (n= 12)

GROUP 1: PBP training + PBP eLearning (n= 12)

Allocation

Excluded (n= 1)

Declined to participate (n= 1)

Randomized (n= 47)

Enrolment

Assessed for eligibility (n= 48)
